# Electrostatic Potentials Caused by the Release of Protons from Photoactivated Compound Sodium 2-Methoxy-5-nitrophenyl Sulfate at the Surface of Bilayer Lipid Membrane

**DOI:** 10.3390/membranes13080722

**Published:** 2023-08-08

**Authors:** Valerij S. Sokolov, Vsevolod Yu. Tashkin, Darya D. Zykova, Yulia V. Kharitonova, Timur R. Galimzyanov, Oleg V. Batishchev

**Affiliations:** 1 Frumkin Institute of Physical Chemistry and Electrochemistry, Russian Academy of Sciences, Leninsky Prospekt, 31, Moscow 119071, Russiaolegbati@gmail.com (O.V.B.); 2 Moscow Institute of Physics and Technology, Institutski Per., 9, Moscow Region, Dolgoprudny 141701, Russia

**Keywords:** bioenergetics, proton transport, caged proton, interfacial proton diffusion, bilayer lipid membrane, boundary potential, membrane electrostatics, membrane capacitance

## Abstract

Lateral transport and release of protons at the water–membrane interface play crucial roles in cell bioenergetics. Therefore, versatile techniques need to be developed for investigating as well as clarifying the main features of these processes at the molecular level. Here, we experimentally measured the kinetics of binding of protons released from the photoactivated compound sodium 2-methoxy-5-nitrophenyl sulfate (MNPS) at the surface of a bilayer lipid membrane (BLM). We developed a theoretical model of this process describing the damage of MNPS coupled with the release of the protons at the membrane surface, as well as the exchange of MNPS molecules and protons between the membrane and solution. We found that the total change in the boundary potential difference across the membrane, ∆ϕ_b_, is the sum of opposing effects of adsorption of MNPS anions and release of protons at the membrane–water interface. Steady-state change in the ∆ϕ_b_ due to protons decreased with the concentration of the buffer and increased with the pH of the solution. The change in the concentration of protons evaluated from measurements of ∆ϕ_b_ was close to that in the unstirred water layer near the BLM. This result, as well as rate constants of the proton exchange between the membrane and the bulk solution, indicated that the rate-limiting step of the proton surface to bulk release is the change in the concentration of protons in the unstirred layer. This means that the protons released from MNPS remain in equilibrium between the BLM surface and an adjacent water layer.

## 1. Introduction

**Figure 1:**
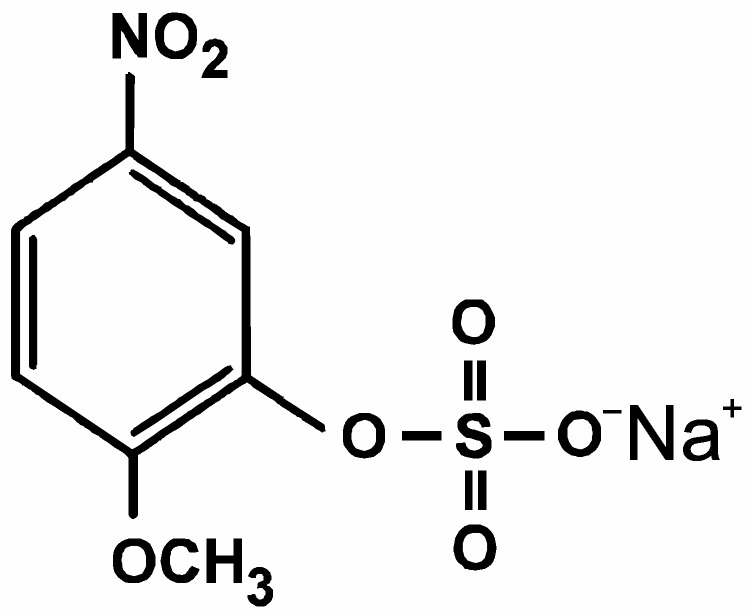
The structure of MNPS.

Interfacial protons at biological membranes play a crucial role in many bioenergetic processes and activity of membrane proteins [1,2]. These surface protons exchange with the bulk, overcoming a high energy barrier formed by water molecules oriented at the membrane–water interface [3,4,5,6]. Therefore, transfer of protons between donor and acceptor molecules in the membrane can proceed along the surface of the membrane instead of bulk water [7,8,9]. This mechanism is essential for the functioning of membrane transporters, where the membrane can serve as a proton-collecting antenna [1,2]. A key question in testing this mechanism is how to evaluate the height of the energy barrier for proton surface to bulk release. This height has been estimated theoretically either by electrostatic models [10] or by molecular dynamics simulations [11]. For experimental values, the kinetics of the exchange of protons between the membrane surface and water is investigated using pH-sensitive fluorescent probes [7,8,9]. The height of the barrier varies depending on experimental conditions and spans from 6 k_B_T for the octanol–water interface [6] to 30 k_B_T in the case of bilayer lipid membranes (BLMs) [7,12]. More easy and rapid estimations of interfacial protons can be achieved by direct electric measurements of the membrane capacitance and electrostatic potentials. Recently, we have observed changes in these values caused by a fast light-driven release of protons from sodium 2-methoxy-5-nitrophenyl sulfate (MNPS) (Figure 1) at the membrane surface that has relaxed in a timescale of about one minute [13]. Study of the kinetics of such slow relaxation can be informative for better understanding of processes coupled with light-driven release of protons from photosensitive compounds. For such investigations, we used the intramembrane field compensation (IFC) method, which allows one to detect the adsorption of charged or dipole molecules at the membrane, as well as their damage by the chemical reactions [14,15,16]. Here, we studied changes in the boundary potential at the membrane interface resulting from the adsorption and photolysis of MNPS molecules. We determined a contribution of the proton binding and the influence of the light intensity, buffer concentration, and pH of the solution.

## 2. Materials and Methods

Bilayer lipid membranes were formed by the Mueller–Rudin technique [17] at a 0.8 mm aperture in a septum separating two 2 mL compartments of a Teflon cell. For BLM formation, 1,2-diphytanoyl-sn-glycero-3-phosphocholine (Avanti Polar Lipids, Alabaster, AL, USA) was dissolved in n-decane (Sigma-Aldrich, St. Louis, MO, USA) to a final concentration of 15 mg/mL. This lipid is chemically stable, in contrast to natural lipids with unsaturated hydrocarbon tails, thus allowing long-lasting experiments with UV illumination without membrane damage. The cell had two windows: one was for the visual control of the BLM and the other was for illuminating. Compartments were filled with 20 mM KCl, 0.2 mM HEPES, 0.2 mM TRIS, and 0.2 mM citric acid (all from Sigma-Aldrich, St. Louis, MO, USA) in double-distilled water and continuously stirred during experiments. Sodium 2-methoxy-5-nitrophenyl sulfate was synthesized in the Zelinsky Institute of Organic Chemistry, Russian Academy of Sciences. Solution of MNPS was added to the distant (relative to the light source) compartment of the cell. To illuminate the BLM, a UV LED (wavelength 375 nm, maximum electric power 3 W) was used. The intensity of the illumination was varied in the range 0.1–0.8 W by changing the voltage applied to the LED. 

For electrical measurements at the BLM, we used a pair of Ag/AgCl electrodes. Electrodes contacted the working buffer solution in the cell through plastic tips, which bottom part was filled with agar, prepared in the solution of 0.1 M KCl in double-distilled water. Electric current was converted to voltage by a Keithley-427 current amplifier (Cleveland, OH, USA) and further analyzed with self-made software. To measure the boundary potential difference (∆ϕ_b_) across the BLM, we used the IFC method ([13], see also reviews [14,15]). This method allowed measuring the difference in boundary potentials between two sides of the BLM after the addition of charged substances or the appearance of charge due to chemical reactions at the one side of the membrane. To measure the open circuit potential (OCP), we added to the lipid solution 10^−5^ M of ionophore pentachlorophenol (PCP) (Sigma-Aldrich, St. Louis, MO, USA).

## 3. Results

**Figure 2:**
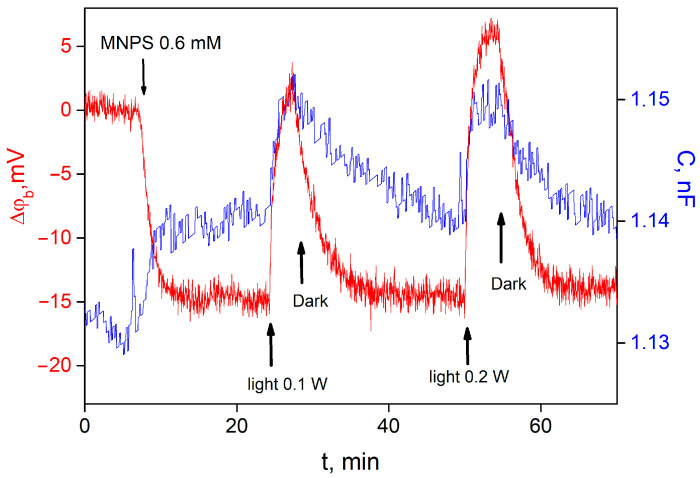
Kinetics of changes in ∆ϕ_b_ (red curve, left scale) and membrane capacitance (blue curve, right scale) due to adsorption of MNPS and illumination with a power of 0.1 W and 0.2 W. Working buffer solution contained 20 mM KCl, 0.2 mM citric acid, 0.2 mM HEPES, 0.2 mM TRIS, pH 8.0.

MNPS is a well-known source of protons that are released after a flash of UV light. Recently, we have demonstrated that MNPS can bond to a lipid membrane and change its boundary potential as well as electrical capacitance under illumination [13]. Addition of MNPS into the water solution at one side of the BLM led to a boundary potential difference across the membrane, the sign of which corresponded to the adsorption of MNPS anions on the membrane. Illumination of the BLM with adsorbed MNPS molecules led to a reversible change in the ∆ϕ_b_ and the membrane electrical capacitance with the magnitude depending on the light intensity (Figure 2). An increase in the intensity of the illumination raised the magnitude and the rate of ∆ϕ_b_ changes.

**Figure 3:**
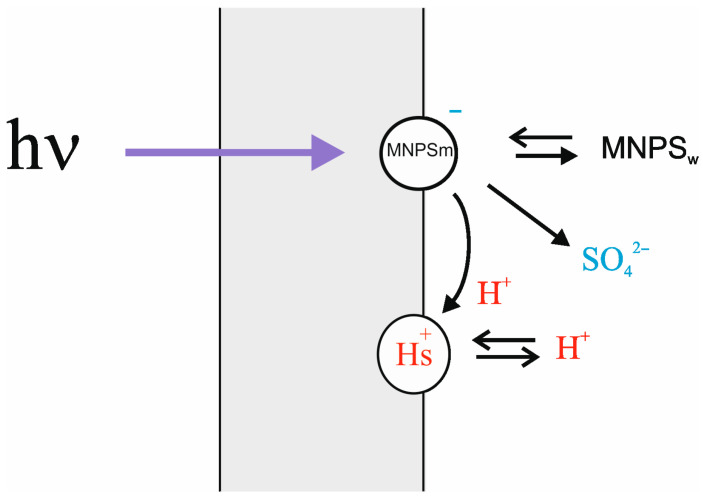
Model of adsorption and transfer of protons and anions by the illumination-induced damage of MNPS at the surface of the BLM.

There are two processes causing the change in ∆ϕ_b_ due to illumination: damage of the MNPS anions at the membrane surface and binding of released protons [13]. To extract the effect of protons from the total change in ∆ϕ_b_, a simple theoretical model was applied (see Appendix A). According to the model, the change in ∆ϕ_b_ under illumination is a result of a decrease in the number of MNPS anions at the membrane surface with a simultaneous increase in the amount of released protons. The model also accounts for the exchange of protons and MNPS anions between the BLM and the bulk water solution. The scheme of processes is shown in Figure 3. 

Based on the model, kinetics of the ∆ϕ_b_ change can be fitted by two exponential functions using four independent parameters: rate constants *k_d_* and *k_dh_* of the desorption from the BLM of MNPS anions and protons, respectively, the rate *k* of the damage of MNPS at the BLM, and the coefficient *B*, which determines the ratio of contributions to the change in ∆ϕ_b_ from the proton binding and MNPS damage. The ratio of these steady-state changes is equal to $B^{*}=B\frac{k_{d}}{k_{dh}}$ (see Appendix A).

**Figure 4:**
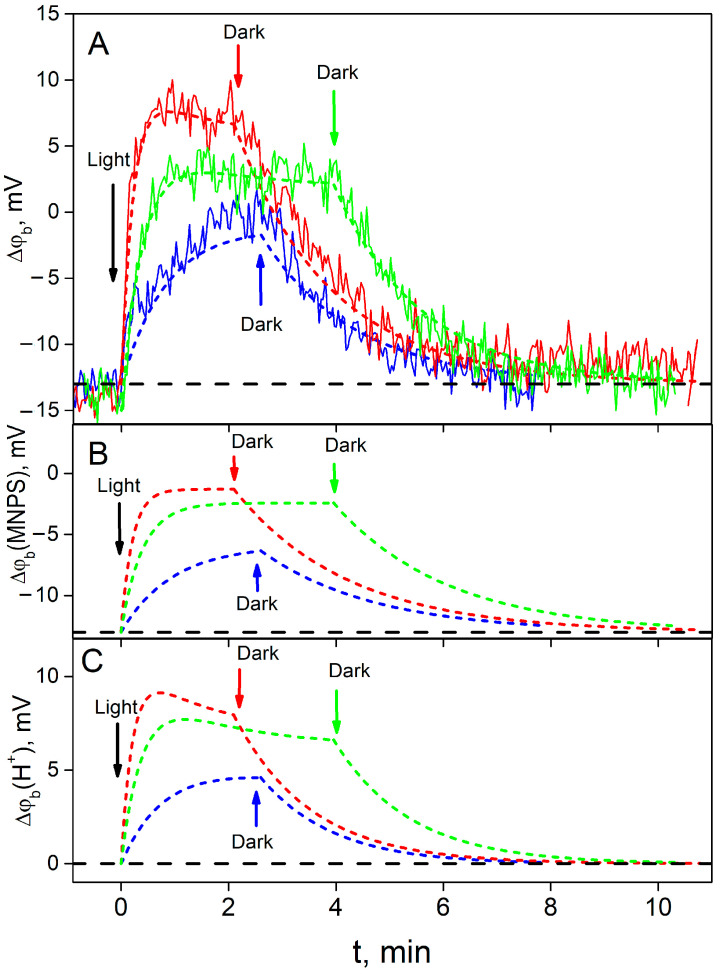
Typical kinetics of ∆ϕ_b_ during illumination with various intensities and its restoration in the dark. Blue, green, and red colors correspond to electric power of 0.1 W, 0.4 W, and 0.8 W, respectively. (**A**) Solid lines are experimental traces; dashed ones are fits by the theoretical model; (**B**) calculated contribution to ∆ϕ_b_ by MNPS; (**C**) calculated contributions to ∆ϕ_b_ by protons. Dashed lines are plotted for the light and dark phases according to the equations: (**A**) ϕ_ads_ × ϕ_rel_(t) (Equations (A10) and (A18)), (**B**) ϕ_ads_ × ϕ_MNPS_(t) (Equations (A19) and (A21)), (**C**) ϕ_ads_ × ϕ_H_(t) (Equations (A20) and (A22)). Parameters of the equations: ϕ_ads_ = –13 mV, *k_dh_* = 0.71 min^−1^, *k_d_* = 0.47 min^−1^, *B* = 0.90, *k_p_* = *k × P*, where *P* is the power of the illumination, *k* = 5.4 min^−1^ W^−1^. Black dashed lines show the initial value of ∆ϕ_b_ before the illumination, ∆ϕ_b_ = ϕ_ads_. The solution contained 20 mM KCl, 0.2 mM citric acid, 0.2 mM HEPES, and 0.2 mM TRIS, pH = 8. The concentration of MNPS was 0.6 mM.

Typical experimental kinetics of the ∆ϕ_b_ change for various light intensities and their fit by the model are shown in Figure 4A. Figure 4B,C demonstrate calculated contributions to the total value of ∆ϕ_b_ from the MNPS and proton binding at the BLM, respectively. As one can see, at the beginning (t = 0), ∆ϕ_b_ was equal to the part produced by the binding of MNPS anions (compare Figure 4A and Figure 4B). After the illumination was switched on, the ∆ϕ_b_ value reflected the sum of a decrease in the number of MNPS anions (Figure 4B) and an increase in the amount of released protons (Figure 4C). The steady-state amount of MNPS anions at the BLM is considerably less than the initial one. The contribution of protons to the ∆ϕ_b_ (Figure 4C) for the high light intensity is non-monotonic and could be described by two exponential functions.

**Figure 5:**
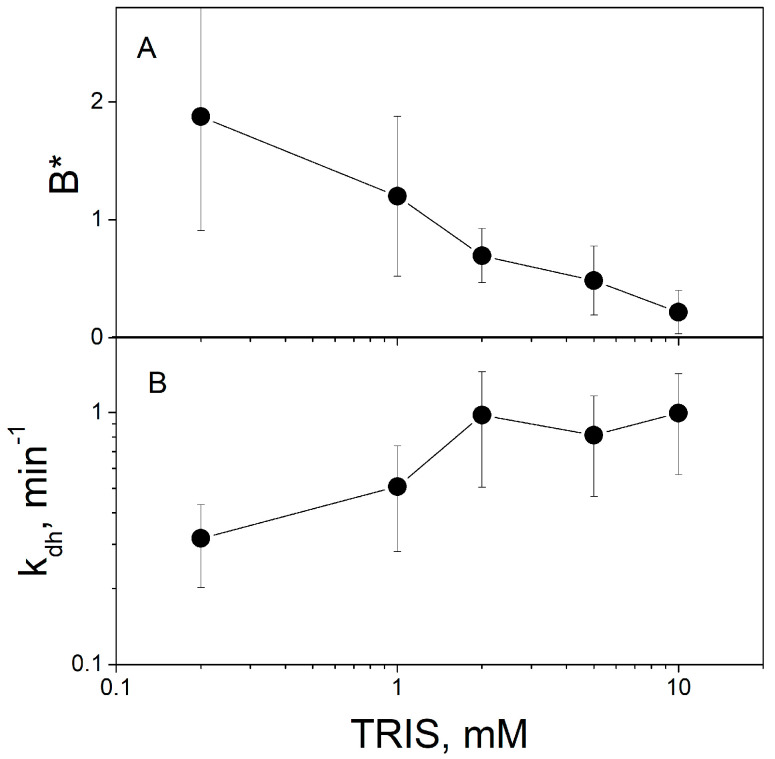
Influence of the concentration of TRIS on the change in ∆ϕ_b_ caused by the illumination of the BLM with adsorbed MNPS. (**A**) Parameter B* indicates the contribution of protons to the steady-state value of ∆ϕ_b_. (**B**) Rate constant of the proton transfer from the membrane, *k_dh_*. The solution contained 20 mM KCl, 0.2 mM citric acid, 0.2 mM HEPES, and the indicated amount of TRIS, pH = 8. The concentration of MNPS and the electrical power of the illumination were 0.6 mM and 0.4 W, respectively.

Contribution of released protons to ∆ϕ_b_ should depend on the concentration of the buffer and pH of the solution. An increase in the buffer concentration reduces the amount of non-equilibrium protons bound to the BLM and the respective part of the ∆ϕ_b_ change [7,9]. Figure 5 demonstrates that the increase in the TRIS concentration decreases the parameter B* (ratio of steady-state ∆ϕ_b_ values caused by protons and MNPS anions) determined by the fitting of the experimental data (Figure 5A) and increases the rate constant *k_dh_* (Figure 5B). 

**Figure 6:**
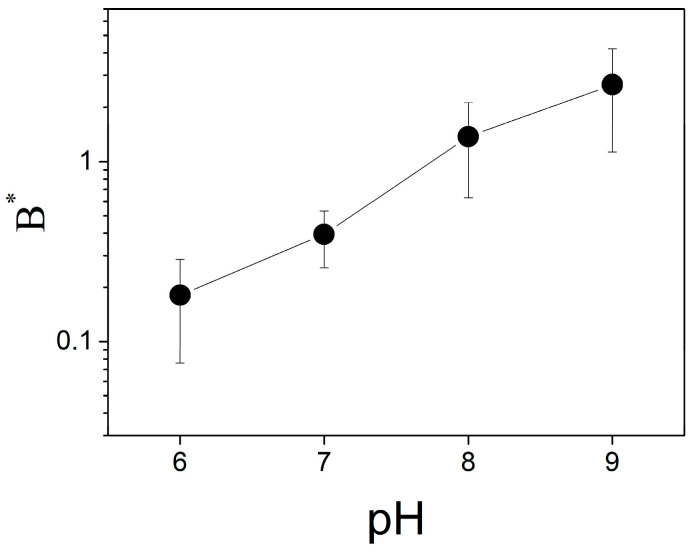
pH dependence of the parameter B*. The solution contained 20 mM KCl, 0.2 mM citric acid, 0.2 mM TRIS, 0.2 mM HEPES. The concentration of MNPS and the electrical power of the illumination were 0.6 mM and 0.4 W, respectively.

The change in ∆ϕ_b_ due to binding of protons depends on the pH of the solution (Figure 6). The parameter B* increases with the pH in the entire range of the measurements, from 6.0 to 9.0. It means that the decrease in pH attenuates the ∆ϕ_b_ change due to bound protons similarly to the effect of the buffer.

**Figure 7:**
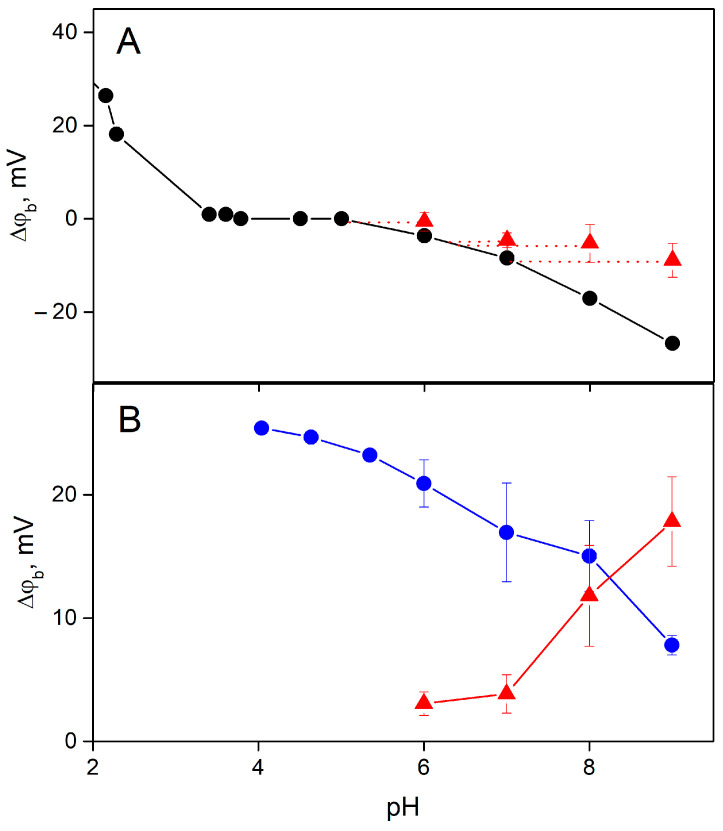
pH dependence of the ∆ϕ_b_ change. (**A**) Dependence of the equilibrium ∆ϕ_b_ on the change in pH of the solution at one side of the membrane (black symbols). The ∆ϕ_b_ value at pH 5 is taken as zero. (**B**) pH dependence of the ∆ϕ_b_ change caused by the adsorption of MNPS (blue symbols). Red symbols designate the change in ∆ϕ_b_ caused by the release of protons from MNPS molecules. The concentration of MNPS and the electrical power of the illumination were 0.6 mM and 0.4 W, respectively. The solution contained 20 mM KCl, 0.2 mM citric acid, 0.2 mM TRIS, 0.2 mM HEPES.

The pH dependence of the ∆ϕ_b_ part caused by protons is determined by the change in the surface charge of the BLM, resulting from the shift in the acid–base dissociation of titratable groups in the polar part of lipids. To study this dependence, we measured the equilibrium change in ∆ϕ_b_ by adding a small amount of HCl or KOH in the solution at one side of the BLM. This dependence is shown in Figure 7A. We observed a similar dependence earlier [18]. Like in [18], the absolute value of ∆ϕ_b_ was taken to be zero at pH 5, because ζ-potential measurements indicate zero surface charge of the BLM at this pH value. We observed changes in ∆ϕ_b_ under illumination at high pH values, where the surface charge of the BLM was negative and release of protons increased the ∆ϕ_b_ value (Figure 7A). The pH dependence of the equilibrium value of ∆ϕ_b_ can be used for evaluating the relative change in the concentration of protons released at the BLM surface. In addition to this curve, we determined the dependence of the ∆ϕ_b_ change due to adsorption of the MNPS on the BLM. As follows from Figure 7B, this part of ∆ϕ_b_ decreased with pH, which could be explained by electrostatic interactions of MNPS anions with the BLM, the negative charge of which increased with pH. 

Release of protons from MNPS decreased pH at the membrane surface that resulted in the ∆ϕ_b_ increase. The total change in ∆ϕ_b_ is a product of boundary potential difference resulting from the MNPS adsorption (Figure 7B, blue symbols) and the coefficient B*, which determines the relative contribution of protons to ∆ϕ_b_ (Figure 6). The proton-related part of ∆ϕ_b_ is plotted in Figure 7B by red symbols. If we add this value to the equilibrium ∆ϕ_b_ (Figure 7A, red symbols) and plot a horizontal line up to the intersection with the pH dependence of equilibrium ∆ϕ_b_ (Figure 7A, black curve), we should obtain the correspondence between equilibrium pH at the membrane surface and change in pH resulting from the illumination. 

**Figure 8:**
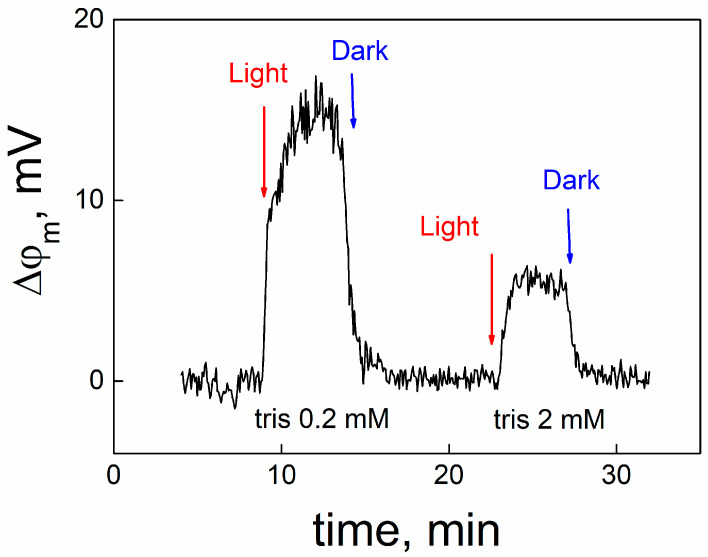
Typical kinetics of the change in the potential of open circuit caused by the release of protons from MNPS at one side of the BLM. Illumination power was 0.4 W. The solution contained 20 mM KCl, 0.2 mM citric acid, TRIS (concentration is indicated in the figure), 0.2 mM HEPES, 10^−5^ M PCP, pH 8. MNPS at a concentration of 0.3 mM was added into the solution at one side of the BLM.

Release of protons from MNPS into the unstirred layer of the solution adjacent to the membrane can be measured by another method, described in [19,20]. We adsorbed MNPS at one side of the BLM, which conducted protons due to the presence of protonophore pentachlorophenol, and measured the open circuit potential with an electrometer. Typical kinetics of the change in OCP under illumination and its following restoration in the dark are presented in Figure 8. 

**Figure 9:**
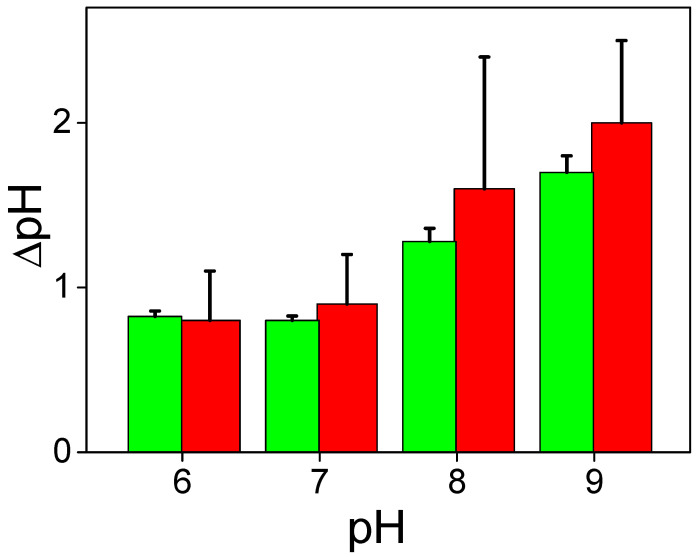
Comparison of the change in the concentration of protons released from MNPS at the surface of the BLM (red bars) and in the unstirred layer of the solution adjacent to the membrane (green bars) at various values of pH of the solution. The solution contained 20 mM KCl, 0.2 mM citric acid, 0.2 mM TRIS, 0.2 mM HEPES. MNPS at a concentration of 0.3 mM was added into the solution at one side of the BLM, the power of illumination was 0.4 W. The details are in the text.

After the beginning of the illumination, the OCP changed due to release of protons from MNPS into the unstirred layer near the BLM. The termination of the illumination led to restoration of the OCP to its initial value. The increase in the concentration of buffer in the solution suppressed the change in OCP caused by illumination. Similar experiments have been performed earlier, when a BLM with adsorbed MNPS was illuminated by a UV light flash [21]. To determine the shift in pH in the unstirred layer, we performed calibration measurements, when the pH value was changed in the bulk water solution by adding HCl into one compartment of the cell. The pH shift in the unstirred layer of the water solution was compared with the change in pH at the membrane surface determined by the ∆ϕ_b_ measurements (Figure 9). This comparison showed that pH shifts determined by these two methods almost coincided in the whole range of pH. 

**Figure 10:**
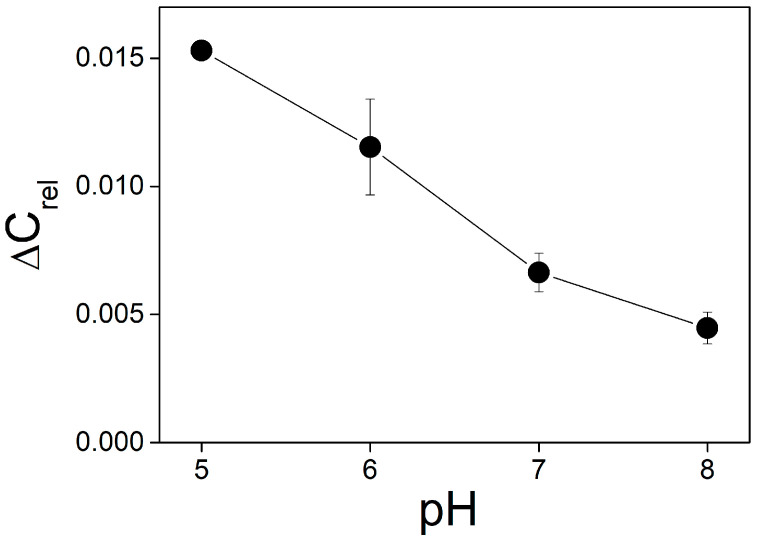
Dependence of the relative change in the membrane capacitance on pH. The solution contained 20 mM KCl, 0.2 mM citric acid, 0.2 mM TRIS, 0.2 mM HEPES. MNPS at a concentration of 0.3 mM was added into the solution at one side of the BLM, the power of illumination was 0.4 W.

Illumination of the BLM with adsorbed molecules of MNPS led not only to a change in ∆ϕ_b_ and pH in the unstirred layer but also to an increase of the membrane capacitance. Like changes in ∆ϕ_b_, the light-induced change in the capacitance depended on the concentration of MNPS, intensity of the illumination, and pH of the solution. In contrast to the changes in ∆ϕ_b_, the change in the capacitance decreased with pH (Figure 10).

## 4. Discussion

Measurements of the kinetics of ∆ϕ_b_ and OCP showed that the total change in ∆ϕ_b_ consisted of two parts connected with the amount of MNPS anions and protons at the surface of the BLM. The contribution corresponding to protons was determined using the simple model accounting for MNPS photolysis and the exchange of MNPS anions and protons between the BLM surface and the bulk solution (see Appendix A). The part of ∆ϕ_b_ caused by protons increases with the MNPS concentration and light intensity, decreases with the concentration of buffer, and depends on pH. Changes in the relative concentration of protons released by the photolysis of MNPS at the surface of the BLM and in the unstirred layer near the membrane were very close. This allows us to conclude that protons released from MNPS molecules remain in an equilibrium between the BLM surface and the adjacent water layer. Therefore, the energy barrier for proton transfer between the lipid membrane and water has a negligible effect on the distribution of protons between the BLM and water. This can be explained by a fast transfer over the barrier at the membrane–water interface and a slow diffusion in the unstirred layer. The coincidence of proton concentration shifts at the membrane surface and in water (Figure 9) indicates that the proton transfer is much faster than diffusion. 

The conclusion that the diffusion of protons in water is the rate-limiting step of the ∆ϕ_b_ change is supported by the evaluation of the rate of the process. Values of *k_dh_* determined by fitting the experimental data by our theoretical model varied in the range 0.4–2.0 min^−1^ that yielded the lowest time constant of the proton surface to bulk release of about 30 s. OCP measurements characterizing the rate of change in pH in the unstirred layer near the membrane gave a time constant of about 10 s. Nevertheless, all these rates were 1–2 orders of magnitude slower than the corresponding rates found from experiments with fluorescent probes [6,7,9]. The slower rate in our experiments indicates that the rate-limiting process is the change in the proton concentration in the unstirred layer. 

The time of equilibration of the concentration of molecules in the unstirred layer can be roughly evaluated as:

$$\tau =\frac{L^{2}}{D}$$

where *L* is the thickness of the unstirred layer (which is about 10^−2^ cm), *D* is the diffusion coefficient (protons diffuse in the water together with buffer molecules, and the value of *D* for most buffer molecules can be taken approximately as 10^−5^ cm^2^ s^−1^). This yields a time of about 10 s, similar to that determined from our experiment. Perhaps experiments with fluorescent probes give faster rates of the proton surface to bulk release because of the better time resolution of the method or usage of other compounds, such as “Caged-H+”. Values of the rate constant *k_dh_* of the proton desorption from the BLM in our experiments were close, by an order of magnitude, to that of rate constant *k_d_* of the MNPS desorption. This could be explained if we assume that both these values correspond to the diffusion of molecules of buffer or MNPS in the water solution. 

**Figure 11:**
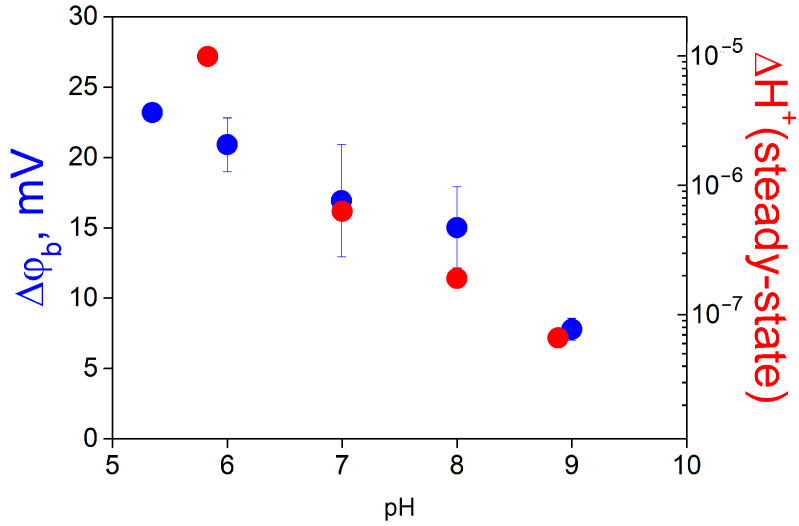
Dependence of the absolute increase in the concentration of protons on pH of the solution (red symbols, right scale). Data were taken from Figure 9. For comparison, the pH dependence of ∆ϕ_b_ caused by the adsorption of MNPS at the BLM is plotted (blue symbols, left scale).

Our results suggest that the main contribution to the change in the ∆ϕ_b_ is determined by the change in pH in the unstirred layer adjacent to the membrane. Surface charge of the BLM should influence the distribution of protons between the membrane and water. Recently, the effect of surface charge on the lateral transfer of protons along the membrane and their release in the bulk solution has been found in experiments with fluorescent probes [12]. The dependence of photoinduced ∆ϕ_b_ change on pH does not reflect the influence of pH on the rate of the reaction of release of protons from MNPS. To find the actual dependence of the reaction on pH, the absolute value of the pH shift caused by the illumination was determined by subtracting the initial concentration of protons from the total values (Figure 7A). These values are plotted in Figure 11. Absolute shifts in the concentration of protons released from MNPS indicate that the reaction of photo release of protons from MNPS does not depend on pH and is determined only by the pH dependence of the adsorption of MNPS anions at the membrane. Note that the pH dependence of the absolute shift in the concentration of protons released from MNPS (Figure 11) is similar to the pH dependence of the membrane capacitance (Figure 10). 

The mechanism of the membrane capacitance change under illumination is unknown. Earlier we observed similar effects, when the BLM was illuminated by a flash of light [13]. There are several factors which could lead to a total change in the capacitance: (i) change in the capacitance of the double layer; (ii) change in the geometric size (thickness or area) of the membrane; or (iii) change in the dielectric permittivity of the water layer near the membrane surface. The first reason can be excluded: the evaluation of the change in the capacitance due to the change in the surface charge using Gouy–Chapman theory gives a value two orders of magnitude lower than the measured one [13]. The most probable mechanism of the capacitance change could be a change in the surface tension at the membrane–water interface due to the damage of MNPS molecules and binding of the protons at the membrane surface. This can influence the membrane geometric size. The most interesting mechanism is the third one. The dielectric permittivity of the water layer at the membrane surface could vary either by the change in the concentration of MNPS molecules and protons at the membrane–water interface or by the reorientation of water molecules near the membrane surface.

The equilibrium dependence of ∆ϕ_b_ on pH (Figure 7) contradicts simple theoretical expectations, if we assume that the surface charge of the BLM formed from PC lipids should be determined only by two charged groups of the phospholipid molecule: negatively charged phosphate (pK_a_ about 2.2) and positively charged choline, which should not dissociate. Therefore, an expected dependence of the surface charge of the BLM on pH should include only the region of the positive charge at low pH, where the phosphate group of the PC molecule becomes neutral. At a pH of around 7, the surface charge of the lipid membrane should be zero. However, the negative surface charge of PC membranes at neutral pH has been observed by various methods [18,22,23] and explained as a result of spontaneous degradation of PC molecules [24], adsorption of OH^–^ or anions of buffer [25], or other inorganic salts [26,27]. The hypothesis about the possible adsorption of OH^–^ on lipid membranes is supported by ζ-potential measurements of gas or oil bubbles that show a negative surface charge at high pH values [28,29,30]. 

An alternative assumption is that the pH of the solution influences the dipole potential of the membrane resulting from the change in the projection of the PC headgroup dipole to the membrane surface [31]. This hypothesis is supported by the corresponding changes in PC molecule shape influencing membrane fusion [32]. The change in dipole potential also reflects the change in the orientation of water molecules bound to polar lipid parts. It is established that the change in entropy of water molecules at the membrane interface relative to the bulk solution gives the main contribution to the energy barrier of proton surface to bulk release [7]. Thus, proton transport in the unstirred layer of the bound water can determine the lateral proton diffusion and its release from the membrane surface to water.

## Data Availability

Data will be available upon reasonable request.

## Appendix A

### Theoretical Model

Processes described in the model are shown in Figure 3. Differential equations describe the damage of MNPS molecules at the membrane, release of protons, and the exchange of MNPS molecules and protons between the membrane and the bulk water solution. For simplicity, reactions and transfer of MNPS and protons are assumed to be independent. This allowed solving the equations with MNPS and protons separately. The kinetics of change in the concentration of MNPS at the membrane is described by the following differential equation:

(A1) $$\frac{d}{dt}{MNPS}_{m}\left(t\right)=-\left(k_{d}+k_{p}\right){MNPS}_{m}\left(t\right)+k_{a}{MNPS}_{w}$$

where *MNPS_m_*(*t*) and *MNPS_w_* are concentrations of MNPS molecules at the membrane and in the bulk solution, correspondingly, *ka* and *k_d_* are rate constants of MNPS adsorption and desorption, *k_p_* is the rate constant of the damage of MNPS at the membrane due to illumination, resulting in its transformation into uncharged product and release of the charged sulfate (SO_4_^2−^) and proton. The initial condition is the equilibrium of MNPS between the membrane and the water solution before the illumination:

(A2) $${MNPS}_{m}\left(0\right)=\frac{k_{a}}{k_{d}}{MNPS}_{w}$$

The solution of this equation is the following exponential function:

(A3) $${MNPS}_{m}\left(t\right)=\frac{k_{a}{MNPS}_{w}\left[k_{d}+k_{p}e^{-\left(k_{d}+k_{p}\right)t}\right]}{k_{d}\left(k_{d}+k_{p}\right)}$$

The kinetics of the change in the concentration of protons bound to the membrane surface *H_s_*(*t*) is described by the following equation:

(A4) $$\frac{d}{dt}H_{s}\left(t\right)=-k_{dh}H_{s}\left(t\right)+k_{ah}H+k_{p}{MNPS}_{m}\left(t\right)$$

where *H* is the concentration of protons in the bulk solution, *k_ah_* and *k_dh_* are rate constants of adsorption and desorption of protons, correspondingly, *MNPS_m_*(*t*) is the function in (A3), found as the solution of Equation (A1). The initial condition is the equilibrium of protons between the surface of the membrane and bulk solution:

(A5) $$H_{s}\left(0\right)=\frac{k_{ah}}{k_{dh}}H$$

The solution of Equation (A4) is the sum of two exponents:

(A6) $$H_{s}\left(t\right)=\frac{k_{ah}}{k_{dh}}H+\frac{k_{a}k_{p}{MNPS}_{w}}{k_{dh}\left(k_{d}+k_{p}\right)}+\frac{k_{a}{k_{p}^{2}MNPS}_{w}}{k_{d}\left(k_{d}+k_{p}\right)\left({k_{dh}-k}_{d}-k_{p}\right)}e^{-\left(k_{d}+k_{p}\right)t}+\frac{k_{a}k_{p}{MNPS}_{w}\left(k_{d}-k_{dh}\right)}{k_{d}k_{dh}\left({k_{dh}-k}_{d}-k_{p}\right)}e^{-k_{dh}t}$$

The ∆ϕ_b_ change measured in the experiment is determined by the concentration of MNPS anions and protons bound to the BLM surface. Protons bound to the BLM before the illumination correspond to the initial ∆ϕ_b_ value taken as zero, therefore, they should be subtracted from the final solution:

(A7) $$\varphi \left(t\right)=-A{MNPS}_{m}\left(t\right)+B\left[H_{s}\left(t\right)-\frac{k_{ah}}{k_{dh}}H\right]$$

where *A* and *B* are coefficients corresponding to the contribution of MNPS anions and protons to the ∆ϕ_b_ change, respectively. A more convenient dimensionless form of the ∆ϕ_b_ change could be found, if we divide Equation (A6) by the ∆ϕ_b_ change, ϕ_ads_, caused by the adsorption of MNPS before the illumination:

(A8) $$\varphi_{ads}=A{MNPS}_{m}\left(0\right)=A\frac{k_{a}}{k_{d}}{MNPS}_{w}$$

Then, the *φ*(*t*) should be replaced by *φ_rel_*(*t*) and *B* by *B_rel_*, according to:

(A9) $$\varphi_{rel}\left(t\right)=\frac{\varphi \left(t\right)}{\varphi_{ads}}=\frac{\varphi \left(t\right)}{A{MNPS}_{m}\left(0\right)},\;B_{rel}=\frac{B}{A}$$

The final solution of equations is:

(A10) $$\varphi_{rel}\left(t\right)=\varphi_{st}+\varphi_{e1}\left(t\right)+\varphi_{e2}\left(t\right)$$

where

(A11) $$\varphi_{st}=\frac{k_{d}\left({-k}_{dh}+B_{rel}k_{p}\right)}{k_{dh}\left(k_{d}+k_{p}\right)}$$

(A12) $$\varphi_{e1}\left(t\right)=\frac{k_{p}\left({-k}_{dh}+B_{rel}k_{p}+k_{d}+k_{p}\right)}{\left(k_{d}+k_{p}\right)\left({k_{dh}-k}_{d}-k_{p}\right)}e^{-\left(k_{d}+k_{p}\right)t}$$

(A13) $$\varphi_{e2}\left(t\right)=\frac{B_{rel}k_{p}\left({-k_{dh}+k}_{d}\right)}{k_{dh}\left({k_{dh}-k}_{d}-k_{p}\right)}e^{-k_{dh}t}$$

The kinetics of restoration of ∆ϕ_b_ after illumination is described similarly to that under illumination with minor changes: the *k_p_* is set to zero and Equation (A1) transforms to:

(A14) $$\frac{d}{dt}{MNPS}_{m}\left(t\right)=-k_{d}{MNPS}_{m}\left(t\right)+k_{a}{MNPS}_{w}$$

Initial conditions correspond to the steady-state concentration of MNPS during the illumination:

(A15) $${MNPS}_{m}\left(0\right)=\frac{k_{a}{MNPS}_{w}}{\left(k_{d}+k_{p}\right)}$$

The solution is:

(A16) $${MNPS}_{m}\left(t\right)=\frac{k_{a}{MNPS}_{w}\left[k_{d}+k_{p}\left(1-e^{-k_{d}t}\right)\right]}{k_{d}\left(k_{d}+k_{p}\right)}$$

Equation (A4) with *k_p_* = 0 transforms to:

(A17) $$\frac{d}{dt}H_{s}\left(t\right)=-k_{dh}H_{s}\left(t\right)+k_{ah}H$$

The initial condition is the steady-state concentration of protons during the illumination:

(A18) $$H_{s}\left(0\right)=\frac{k_{ah}}{k_{dh}}H+\frac{k_{a}k_{p}{MNPS}_{w}}{k_{dh}\left(k_{d}+k_{p}\right)}$$

The solution is:

(A19) $$H_{s}\left(t\right)=\frac{k_{ah}}{k_{dh}}H+\frac{k_{a}k_{p}{MNPS}_{w}}{k_{dh}\left(k_{d}+k_{p}\right)}e^{-k_{dh}t}$$

Similar to Equation (A6), the ∆ϕ_b_ change is described by:

(A20) $$\varphi \left(t\right)=-A\frac{k_{a}{MNPS}_{w}\left[k_{d}+k_{p}\left(1-e^{-k_{d}t}\right)\right]}{k_{d}\left(k_{d}+k_{p}\right)}+B\frac{k_{a}k_{p}{MNPS}_{w}}{k_{dh}\left(k_{d}+k_{p}\right)}e^{-k_{dh}t}$$

The relative change in ∆ϕ_b_ is:

(A21) $$\varphi_{rel}\left(t\right)=-1+\frac{k_{p}e^{-k_{d}t}}{\left(k_{d}+k_{p}\right)}+B_{rel}\frac{k_{d}k_{p}e^{-k_{dh}t}}{k_{dh}\left(k_{d}+k_{p}\right)}$$

To analyze the experimental data, the splitting of the steady-state change in ∆ϕ_b_ into contributions corresponding to the change in the number of MNPS anions and protons is necessary. The non-steady-state contribution of MNPS anions to the ∆ϕ_b_ change during illumination is a single exponential function:

(A22) $$\varphi_{MNPS}\left(t\right)=-\frac{k_{p}}{\left(k_{d}+k_{p}\right)}e^{-\left(k_{d}+k_{p}\right)t}$$

The corresponding contribution to ∆ϕ_b_ from protons is the sum of two exponents:

(A23) $$\varphi_{H}\left(t\right)=\frac{B_{rel}k_{p}^{2}}{\left(k_{d}+k_{p}\right)\left({k_{dh}-k}_{d}-k_{p}\right)}e^{-\left(k_{d}+k_{p}\right)t}+\frac{B_{rel}k_{p}\left({-k_{dh}+k}_{d}\right)}{k_{dh}\left({k_{dh}-k}_{d}-k_{p}\right)}e^{-k_{dh}t}$$

The restoring of the potential after the illumination is a single exponential function for both MNPS anions:

(A24) $$\varphi_{MNPS}\left(t\right)=\frac{k_{p}e^{-k_{d}t}}{\left(k_{d}+k_{p}\right)}$$

and protons:

(A25) $$\varphi_{H}\left(t\right)=B_{rel}\frac{k_{d}k_{p}e^{-k_{dh}t}}{k_{dh}\left(k_{d}+k_{p}\right)}$$

The steady-state relative change in the potential due to illumination is the difference between *φ_st_* and −1 (−1 is the relative potential before the illumination determined only by MNPS anions at the membrane):

(A26) $$∆\varphi_{st}=\varphi_{st}+1=\frac{k_{d}\left({-k}_{dh}+B_{rel}k_{p}\right)}{k_{dh}\left(k_{d}+k_{p}\right)}+1$$

The contribution to this value of MNPS anions is

(A27) $$∆\varphi_{st}\left({MNPS}^{-}\right)=\frac{k_{d}\left({-k}_{dh}\right)}{k_{dh}\left(k_{d}+k_{p}\right)}+1=\frac{k_{p}}{\left(k_{d}+k_{p}\right)}$$

and the corresponding contribution of protons is:

(A28) $$∆\varphi_{st}\left(H^{+}\right)=\frac{k_{d}B_{rel}k_{p}}{k_{dh}\left(k_{d}+k_{p}\right)}$$

The ratio of these contributions, *B**, is

(A29) $$B^{*}=\frac{∆\varphi_{st}\left(H^{+}\right)}{∆\varphi_{st}\left({MNPS}^{-}\right)}=\frac{k_{d}B_{rel}}{k_{dh}}$$
